# Biochemistry of proinflammatory macrophage activation

**DOI:** 10.1007/s00018-018-2784-1

**Published:** 2018-03-03

**Authors:** Yannic Nonnenmacher, Karsten Hiller

**Affiliations:** 1 Department of Bioinformatics and Biochemistry and Braunschweig Integrated Center of Systems Biology (BRICS), Technische Universität Braunschweig, Rebenring 56, 38106 Brunswick, Germany; 2 Computational Biology of Infection Research, Helmholtz Centre for Infection Research, Inhoffenstraße 7, 38124 Brunswick, Germany

**Keywords:** Macrophage, Inflammation, Metabolism, Infection, Itaconate, Succinate

## Abstract

In the last decade, metabolism has been recognized as a major determinant of immunological processes. During an inflammatory response, macrophages undergo striking changes in their metabolism. This metabolic reprogramming is governed by a complex interplay between metabolic enzymes and metabolites of different pathways and represents the basis for proper macrophage function. It is now evident that these changes go far beyond the well-known Warburg effect and the perturbation of metabolic targets is being investigated as a means to treat infections and auto-immune diseases. In the present review, we will aim to provide an overview of the metabolic responses during proinflammatory macrophage activation and show how these changes modulate the immune response.

## Introduction

As a part of the innate immune system, peripheral monocytes are amongst the first responders of an infection, indiscriminately engulfing everything that does not originate from the host organism. Macrophages belong to the group of mononuclear phagocytes and are present in almost every corner of the body. Depending on their exact location in the organism, different types of macrophages, with functions specifically tailored to the tissue they inhabit, have been identified throughout the last century. These types include, e.g., peripheral macrophages in the blood, peritoneal macrophages, pulmonary macrophages, Kupffer cells in the liver, and microglia in the brain [1]. The tissue resident variants can either originate from the proliferation of embryonic progenitor-derived macrophages, or the recruitment and subsequent proliferation of mononuclear cells from the blood. Independent of their origin or localization in the body, the aim during an infection remains the same: to engulf pathogens and present their antigens to members of the adaptive immune system, thereby initiating the immune response. During this process, macrophages also produce and secrete a wide array of cytokines, which further activate fellow immune cells. Depending on the type of threat recognized by the macrophage, these cytokines can be of a pro- or anti-inflammatory nature. The transition of macrophages to an inflammation-promoting phenotype is induced by proinflammatory cytokines as well as pathogen-associated molecular patterns (PAMPs), such as lipopolysaccharide (LPS), which are primarily sensed by members of the Toll-like receptor (TLR) family [2]. Macrophages activated in such a way are often termed classically activated macrophages (CAMs) or M1 macrophages [3]. These cells exhibit a strong bactericidal and phagocytotic potential, and aim to fend off infections in the body. On the other hand, quiescent macrophages can be directed towards an anti-inflammatory phenotype by the action of anti-inflammatory cytokines, parasitic infections, and damage-associated molecular patterns (DAMPs). The resulting cells play an important role in tissue repair and wound healing and are referred to as alternatively activated macrophages (AAMs), or M2 macrophages [3]. M1 macrophages represent a first line of defense, rapidly acting against an arising infection, whereas M2 macrophages exhibit long-term functions in resolving the inflammatory response [4]. Classification into the M1 and M2 phenotype is, however, subject to recent discussion and should be used with caution [5]. In the context of in vitro experiments, M1 usually describes macrophages stimulated with LPS and interferon gamma (or M[LPS + IFNγ]), whereas M2 equates to IL-4 stimulation (or M[IL-4]). Macrophages activated by other factors are mostly classified as M1- or M2-like. Although such a broad classification can be performed based on the response of distinct pro- and anti-inflammatory markers, the overall phenotype of these cells often differs substantially from the M[LPS + IFNγ] and M[IL-4] states [6]. It should also be noted that the environment of macrophages in vivo is presumably too complex to allow for such specific polarization phenotypes, and rather gives rise to several intermediate activation states [7]. Nevertheless, analysis of the M[LPS + IFNγ] or M[LPS] and M[IL-4] cells has proven to be a very useful tool in elucidating the molecular mechanisms that orchestrate the inflammatory response.

Both, classically and alternatively activated macrophages, are not only characterized by the production and upregulation of a distinct set of cytokines and genes, but also by a profound and specific rearrangement of metabolic fluxes. In general, M1 macrophages switch from oxidative phosphorylation (OxPhos) to glycolysis for ATP generation. In turn, M2 cells go the opposite direction with moderate glycolytic and high OxPhos activity [8]. An increasing number of studies currently add more and more metabolic features to the portfolio of each activation state, demonstrating how metabolic changes are not just the result of the inflammatory response, but rather a critical modulator of the entire process. In the following, we will describe which metabolic events take place in the different parts of cellular energy metabolism when macrophages encounter proinflammatory stimuli, and discuss how these processes interfere with the immune response.

## Glycolysis

### Classically activated macrophages rely on glycolysis for energy generation

**Fig. 1:**
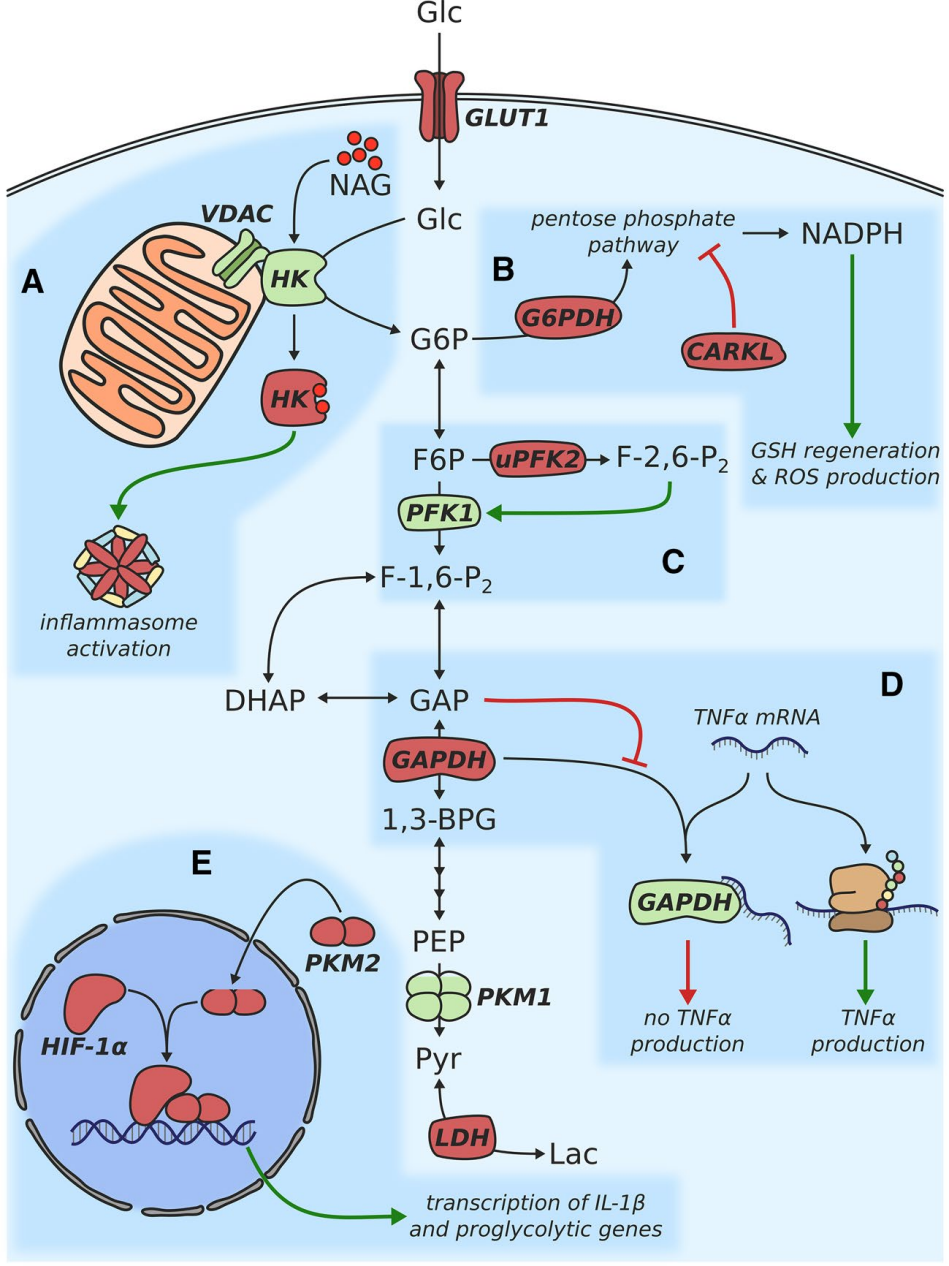
Schematic depiction of metabolic changes associated with glucose metabolism in proinflammatory macrophages

First evidence of metabolic reprogramming in activated macrophages was provided by a study published in 1970 [9]. Gordon C. Hard compared several biochemical parameters of peritoneal macrophages from normal mice and mice infected with *Corynebacterium ovis*. He showed that while activated macrophages produced more lactate, oxygen uptake was higher in unstimulated cells. This indicated that activated macrophages mainly rely on aerobic glycolysis (commonly referred to as the Warburg effect [10]) for ATP generation. His findings were confirmed in another study 16 years later, which provided further information on changes in the activity of individual glycolytic enzymes as well as enzymes involved in fatty acid and amino acid metabolism [11]. At the time, however, it could not be determined which factors regulate these metabolic changes and whether altered metabolism plays a role in modulating the inflammatory response. Recent studies have picked up on these questions and made use of modern methods and technologies to shed light on the role of metabolism during inflammation. In the last decade, many enzymes involved in glycolysis have been found to also act as major players in controlling inflammation. In this section, we will focus on the individual components of glycolysis and discuss how they are involved in the inflammatory process. A schematic overview of these components is also depicted in Fig. 1.

### GLUT1

The uptake of glucose into the cell is generally mediated by carriers of the SLC2 family [12]. In both resting and M(LPS) macrophages, this task is predominantly carried out by the GLUT1 uniporter [13, 14]. Overexpression of GLUT1 in LPS-primed, murine macrophages has been shown to boost glycolytic activity as well as the production of the proinflammatory cytokines IL-6 and TNFα, demonstrating a causal link between glucose metabolism and the immune response [14]. GLUT1 overexpressing cells further exhibited decreased OxPhos activity and accumulated intermediates of the pentose phosphate pathway (PPP)—both of these observations have previously been demonstrated to be characteristic features of M1 polarized macrophages [6]. In contrast, competitive inhibition of glycolysis via 2-deoxyglucose (2-DG) dampened the production of the proinflammatory cytokine IL-1β [15]. Interestingly, the connection between glycolytic flux and IL-1β involves action of the transcription factor hypoxia inducible factor-1α (HIF-1α), which is stabilized by accumulation of succinate and the production of mitochondrial ROS (mROS) upon TLR4 stimulation (also see section “Succinic acid”).

### Hexokinase

Once glucose has passed the cytosolic membrane, it is phosphorylated to glucose-6-phosphate to initiate glycolysis—a reaction catalyzed by the hexokinase (HK) enzyme. Besides its enzymatic function, HK was recently found to be a sensor for the bacterial cell wall degradation product *N*-acetylglucosamine (NAG) [16]. Bacterial peptidoglycans are usually sensed via the cytosolic NOD2 receptor which subsequently triggers an inflammatory response. However, this was not the case for NAG, which is able to induce NLRP3 inflammasome activation even when NOD2 is not present [17]. Instead, NAG binds to HK in a competitive manner, leading to its inactivation and dissociation from the mitochondrial outer membrane (Fig. 1a) [16]. This dissociation alone is sufficient to trigger inflammasome activation, as was shown by the application of a peptide competing with HK for binding to the voltage-dependent anion channel (VDAC) located on the surface of mitochondria. Interestingly, in this study, 2-DG-mediated inhibition of HK resulted in increased IL-1β production which is in contrast to previous studies demonstrating a dampened IL-1β response when the same inhibitor was applied [15, 18]. This ambiguity can possibly be explained by differences in stimulation conditions, 2-DG concentration, cell viability, or the timing of hexokinase inhibition [19]. Nonetheless, this example clearly demonstrates that further research is required to identify the determinants that eventually lead to seemingly opposite results when applying the same treatment.

### 6-Phosphofructo-2-kinase/fructose-2,6-biphosphatase

The next enzyme in the glycolytic chain associated with inflammatory regulation is the 6-phosphofructo-2-kinase/fructose-2,6-biphosphatase enzyme (PFK2; encoded by the *PFKFB3* gene) which catalyzes the conversion of fructose 6-phosphate (F6P) to fructose-2,6-bisphosphate (F-2,6-P_2_). The latter acts as an allosteric activator of PFK1 and as a repressor of fructose-1,6-bisphosphatase (FBPase), respectively [20]. As a result, increased concentrations of F-2,6-P_2_ potentiate glycolytic flux (Fig. 1c). Upon classical activation or stimulation with TLR-2, -3, -4, or -9 agonists, macrophages were shown to exhibit a switch from the liver form of PFK2 (L-PFK2) to its ubiquitous form (uPFK2) [21]. While the enzymatic activity of L-PFK2 is relatively low, the more active uPFK2 maintains higher F-2,6-P_2_ levels, resulting in increased glycolytic activity. Induction of the uPFK2 isoform has been described under hypoxic conditions, where it is triggered by stabilization of HIF-1α [22]. A similar behavior was observed in response to classical activation [21], where HIF-1α is stabilized by accumulating succinate [15] and increased mitochondrial ROS [23]. Intriguingly, the switch to the uPFK2 isoform still occurred in HIF-1α^−/−^ macrophages, suggesting that uPFK2 expression is regulated through a mechanism independent of HIF-1α. Expression of several inflammatory transcripts (IL-6, IP-10, NOS2, and Arg-1) in macrophages activated with LPS and IFN-γ was also unaffected by the ablation of HIF-1α. A RNAi-mediated knockdown of uPFK2, on the other hand, resulted in decreased expression of the proinflammatory markers NOS2 and COX-2. These results point towards a role for PFK2 in the metabolic activation of proinflammatory macrophages. A recent study confirmed this notion by demonstrating the importance of PFK during viral infections [24]. In this model, PFK-induced glycolytic metabolism supported the phagocytosis and elimination of viruses by macrophages.

### Glyceraldehyde 3-phosphate dehydrogenase

A remarkable, yet unexpected function was recently unveiled for the glyceraldehyde 3-phosphate dehydrogenase (GAPDH) enzyme. Besides its well-known catalytic function in glycolysis, it was shown that GAPDH can repress translation of the proinflammatory cytokine TNFα by binding to its mRNA (Fig. 1d) [25]. Although GAPDH was known to act as a RNA-binding protein (especially of AU-rich elements) [26], its role during the proinflammatory response was previously unheard of. Millet and colleagues demonstrated an inverse relationship between glycolytic activity and GAPDH-mediated repression of TNFα expression, suggesting that GAPDH is only able to carry out one of its two tasks at a time [25]. Indeed, the previous studies have shown that the binding of GAPDH to generic AU-rich elements is inhibited by its substrate glyceraldehyde 3-phosphate [27] and its cofactor NAD^+^ [26]. Mechanistically, GAPDH was found to inhibit TNFα translation by binding to the 3′ untranslated region of TNF mRNA. This binding could be reversed by either knocking down GAPDH or increasing glycolytic flux via treatment with insulin or other chemical effectors. The observed effects on TNFα production under these conditions were, however, relatively small as compared to mechanisms regulating the transcription of TNF mRNA [28]. Accordingly, the described mechanism seems to be a way of fine-tuning the inflammatory response, rather than a critical determinant of TNF expression. It should also be noted that the regulatory activity of GAPDH might be especially relevant during the first 4 h after the proinflammatory stimulus, since another study found no connection between glycolytic activity and TNFα production at later timepoints [15]. The same mechanism was recently also described for the regulation of IFN-γ by GAPDH in T cells [29] and it will be interesting to see if a similar kind of regulation will hold true for other metabolic enzymes in the future.

### Pyruvate kinase

The last, rate-limiting step of glycolysis is represented by the conversion of phosphoenolpyruvate to pyruvate—a reaction catalyzed by the pyruvate kinase (PK) enzyme. In almost all cell types (with the exception of liver and red blood cells), this enzyme exists in two different isoforms, termed PKM1 and PKM2, both of which are encoded by the same gene via alternative splicing [30, 31]. While PKM1 is mostly present as a homotetramer with high enzymatic activity, the PKM2 isoform predominantly exists as a monomer or dimer. Although mono- or dimeric PKM2 exerts a lower enzymatic activity, it has been shown to be an important factor driving glycolysis and lactate production in cancer cells [32]. This effect is explained by the transition of dimeric PKM2 from the cytosol to the nucleus, where it acts as a transcription factor to promote the expression of glycolytic enzymes by interacting with HIF-1α [33]. Interestingly, this switch from PKM1 to PKM2, as well as translocation of PKM2 to the nucleus was also observed upon TLR4 activation of murine macrophages [34]. In response to LPS, nuclear, dimeric PKM2 was shown to form a complex with HIF-1α that enhances transcription of IL-1β by binding to its promotor (Fig. 1e). When the emerging PKM2 was, however, forced into the enzymatically active tetrameric state via treatment with small-molecule activators, the proinflammatory response to LPS was dampened. The inhibitory effect of PKM2 activation on the production of IL-1β was also confirmed in macrophages infected with *M. tuberculosis* in vitro. In this setup, small-molecule-induced PKM2 tetramerization, additionally, boosted levels of IL-10, indicating a role of PKM2 in macrophage polarization. On the metabolic level, chemical tetramerization of PKM2 impaired glycolysis and succinate accumulation—two metabolic events commonly upregulated during proinflammatory macrophage activation (also see section “Succinic acid”). This effect on both glycolysis and succinate accumulation appears to be dependent on HIF-1α, since isoform-specific deletion of PKM2 decreased levels of HIF-1α and the expression of its downstream targets IL-1β and lactate dehydrogenase A (LDHA). Another LPS-induced event that could be alleviated by activation of PKM2 was the accumulation of the PPP-intermediate ribose 5-phosphate, suggesting a role for PKM2 in regulating carbon flux between glycolysis and the PPP.

## Pentose phosphate pathway

### Production of ROS and RNS

An upregulation of the pentose phosphate pathway (PPP) in activated murine macrophages was already described in the late 1970s [35]. Another study confirmed this finding and demonstrated that the expression of glucose-6-phosphate dehydrogenase (G6PDH), the first and rate-limiting enzyme of the PPP, is enhanced in response to LPS (Fig. 1b) [36]. Although these early studies already suggested an involvement of the PPP in the inflammatory response, the molecular mechanisms underlying this metabolic adaptation remained elusive. The two main tasks of the PPP are (1) to provide precursors for nucleotide and amino acid synthesis and (2) to generate NADPH, which is required for the synthesis of fatty acids as well as for the regeneration of glutathione (GSH) [37]. While the regeneration of GSH serves as a means to alleviate oxidative stress, activated macrophages also use PPP-derived NADPH to generate reactive oxygen species (ROS). The high oxidation potential of these ROS is exploited to kill pathogens in the phagosome [38, 39], but ROS can also influence various cellular processes by oxidizing redox-sensitive residues of proteins [40, 41]. The controlled, enzymatic generation of ROS is mainly mediated by seven different isoforms of the NADPH oxidase (NOX) [42, 43]. Knockout of NOX1 and NOX2 has recently been shown to impair the monocyte-to-macrophage differentiation as well as M2 polarization, demonstrating the importance of controlled ROS production for macrophage function [44]. In addition to ROS, reactive nitrogen species (RNS) such as nitric oxide (NO) have been found to amplify the proinflammatory phenotype [45]. In macrophages, NO can be produced by the inducible nitric oxide synthase (iNOS or NOS2), which generates NO as a byproduct during the conversion of arginine to citrulline [46]. Upregulation of this reaction has already been found to occur in LPS and IFNγ stimulated macrophages in the 1980s [47, 48], and was subsequently established as a clear marker of M1 polarization [49]. Mechanistically, NO induces a nitrosylation of iron–sulfur proteins, leading to their inactivation [50]. In the proinflammatory macrophage, this mechanism decreases the activity of two members of the mitochondrial electron transport chain: complex I [51] and cytochrome *c* oxidase [52]. Accordingly, NO is one of the factors which push activated macrophages towards a metabolic phenotype characterized by low mitochondrial OxPhos and high glycolytic activity. A recent study performed in a murine model found that a wide range of enzymes can be modified via the S-nitrosylation of cysteine residues [53]. These enzymes are found in almost all parts of central carbon metabolism including glycolysis, gluconeogenesis, tricarboxylic acid cycle, and oxidative phosphorylation, highlighting the importance of NO as a regulator of metabolism. Interestingly, both isocitrate dehydrogenase (IDH) and succinate dehydrogenase (SDH, complex II of the ETC) were also among the identified proteins [53]. A decrease in activity of both of these enzymes has recently been discovered to play a major role for the course and severity of an inflammatory response in macrophages (also see sections “Succinate accumulation” and “Metabolic reprogramming assists in itaconic acid production”) [15, 54].

In contrast to M1 polarized cells, M2 macrophages mostly convert arginine to ornithine instead, which is used to synthesize polyamines that aid in tissue-repair functions. This differential metabolism of arginine was reported to be dependent on the activity of different isoforms of HIF [55]. While the HIF-1α isoform promotes the synthesis of NO and M1 polarization, the HIF-2α isoform induces arginase activity and M2 polarization. The dependence of both NOX and iNOS on the cofactor NADPH makes the PPP a key regulator for the production of ROS and RNS and, thus, the inflammatory response.

The PPP can be separated into two distinct phases. In a first series of reactions, glucose-6-phosphate (G6P) is converted to ribulose 5-phosphate (oxidative phase). During this phase, 2 mol of NADPH are produced per mol of G6P in the reactions catalyzed by the G6PD and 6-phosphogluconate dehydrogenase (PGD) enzymes. The second, non-oxidative branch of the PPP consists of a series of carbon-backbone rearrangements which supply precursors for amino acid and nucleotide synthesis or feed carbon atoms back into glycolysis. Interestingly, the expression levels of G6PD and PGD were found to be elevated [36, 54, 56], and the relative carbon flux through the oxidative part of the PPP was increased in activated macrophages [54]. Overexpression of G6PD stimulated, whereas its siRNA-mediated knockdown attenuated, the expression of several proinflammatory cytokines, including IL-6, IL-1β, MCP-1, and TNFα [56]. The levels of both NOX2 and iNOS as well as ROS production were also correlated to the abundance of G6PD protein. This finding supports the hypothesis that cytokine expression is regulated by ROS/RNS, which is further confirmed by the fact that the observed effects could be partially reversed by the action of radical scavengers.

### Role of CARKL

Haschemi et al. have shown that expression of the carbohydrate kinase-like protein (CARKL) rapidly decreases upon TLR4 activation of both human and murine macrophages [57]. CARKL was found to act as a non-protein kinase, catalyzing the formation of sedoheptulose-7-phosphate from sedoheptulose—an orphan reaction of the PPP (Fig. 1b). Loss of this enzyme was shown to be one of the factors that contribute to both cytokine production and metabolic reprogramming in LPS-activated macrophages. Overexpression of CARKL limited flux though the PPP and induced a striking shift towards a more oxidized redox state, manifesting itself in increased NAD/NADH and GSSG/GSH ratios. These shifts seem to be the result of limited NADPH availability, which is vital for the reduction of redox couples. Production of superoxide radicals (O_2_^∙−^), a typical feature of proinflammatory macrophages, was also blunted upon CARKL overexpression. Again, NADPH limitation could explain this behavior, given the fact that classically activated macrophages (CAMs) exploit the NADPH oxidase system for ROS production [39, 44, 58, 59]. In activated macrophages, this might imply that the increased production of NADPH via the PPP is not only necessary to produce ROS, but also to ensure a sufficient supply of antioxidants to prevent the adverse effects which the ROS might cause. Overexpression of CARKL further led to a reduction in glycolytic flux, which is usually strongly upregulated in proinflammatory macrophages [15, 21, 34]. The production of key proinflammatory transcripts (i.e., TNFα, IL,-1β, IL-6, and IL-13R) also appeared to be dependent on the absence of CARKL. Concurrently, overexpression of CARKL pushed the expression of anti-inflammatory markers, as for example IL-10 and the IL-4 receptor. Stimulation with agents that drive M2-like polarization, i.e., IL-4 and IL-13, induced a mild boost in CARKL levels, thus even suggesting a regulatory role for CARKL in macrophage polarization. Notably, the CARKL-mediated effects on macrophage metabolism and cytokine production are directly linked to its enzymatic activity, as the effects were not observed in CARKL variants carrying a mutation in the catalytic site of the protein. This study nicely shows how the inflammatory response can be regulated and controlled by modulating metabolic flux.

## Succinic acid

### Succinate accumulation

**Fig. 2:**
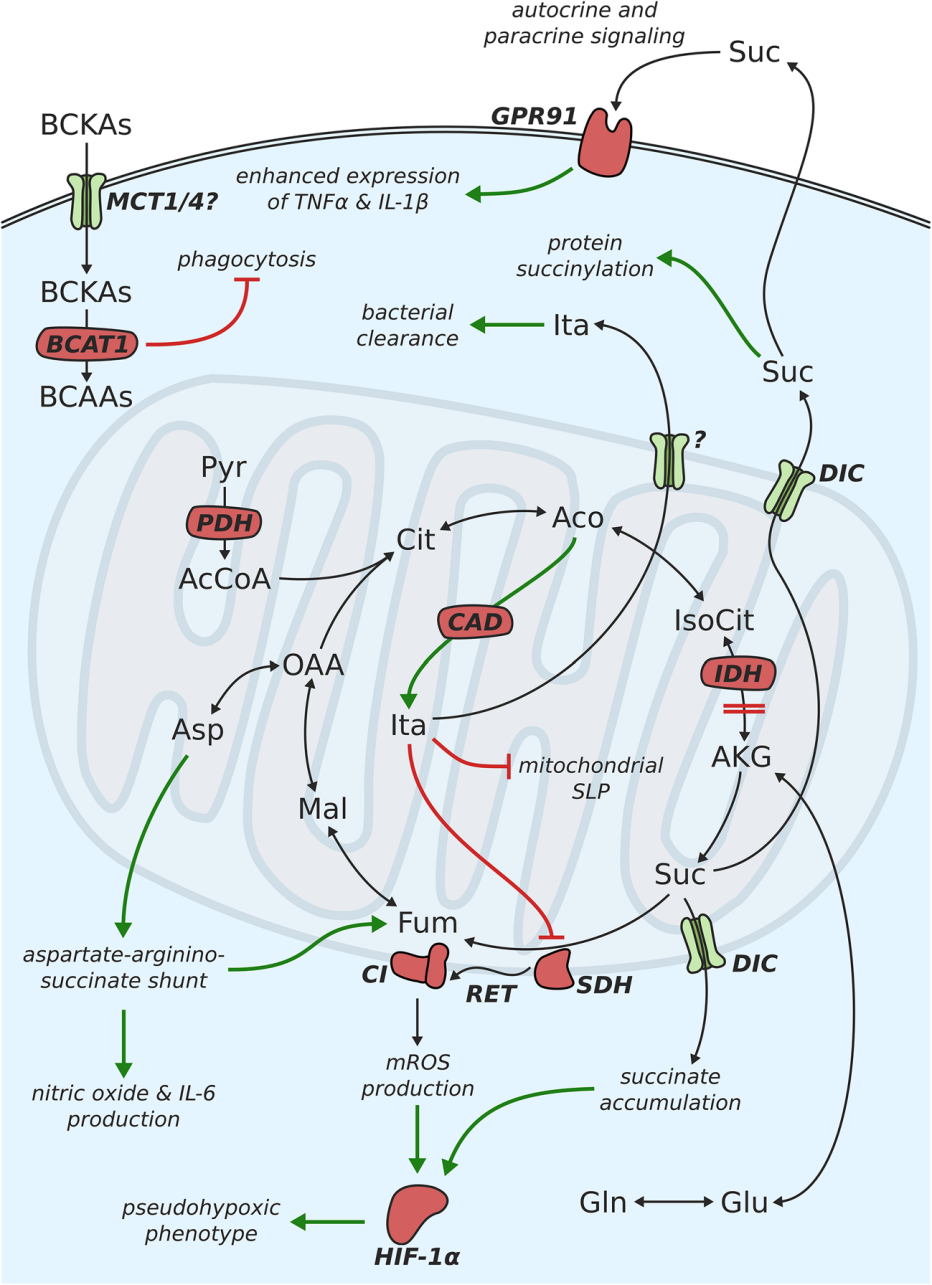
Schematic depiction of metabolic changes associated with mitochondrial metabolism in proinflammatory macrophages

One of the most profound changes in the metabolism of M1-like polarized macrophages is the intracellular accumulation of high amounts of the TCA cycle intermediate succinate [15]. This observation was first made in a study investigating the influence of glycolytic activity on cytokine production. Interestingly, inhibition of glycolysis using 2-DG decreased the levels of both IL-1β production and succinate accumulation, suggesting a functional connection between the two. The previous studies on cancer metabolism have shown that succinate can induce stabilization of the transcription factor HIF-1α [60]. Under normoxic conditions, HIF-1α is hydroxylated by cytosolic HIF-α prolyl hydroxylase (PHD) enzymes and subsequently degraded. During this reaction, α-ketoglutarate is converted to succinate. Accumulating succinate is transported from the mitochondria to the cytosol via the dicarboxylate carrier (DIC). In the cytosol, it induced product inhibition of PHDs, and thus, prevents hydroxylation and degradation of HIF-1α (Fig. 2). In the activated macrophage, HIF-1α then proceeds to push aerobic glycolysis and lactate production, as well as the transcription of IL-1β by binding to its promotor [34]. The intracellular level of succinate is mainly regulated by the activity of SDH. Treatment with the SDH-inhibitor butylmalonate or cell-permeable diethylsuccinate increased intracellular succinate levels and HIF-1α and IL-1β protein abundance in LPS-activated macrophages [15]. The dependence of IL-1β expression on HIF-1α was further demonstrated by the application of HIF-1α^−/−^ macrophages, which showed a dampened response to diethylsuccinate treatment.

### SDH-mediated ROS production

Although many of the effects discussed above can be explained by accumulating succinate, a more recent study highlighted the importance of SDH activity as a main driver of proinflammatory macrophage activation [23]. The authors applied dimethylmalonate (DMM) which is hydrolyzed in the cell to form malonate—a competitive inhibitor of SDH-mediated succinate oxidation [61]. Intriguingly, treatment with DMM increased succinate levels, but failed to stabilize HIF-1α and increase IL-1β expression. This seems especially odd, given that these expected outcomes were observed in a previous study using the SDH-inhibitor butylmalonate [15]. In contrast, DMM decreased LPS-induced IL-1β mRNA, pro-IL-1β, and HIF-1α protein (but not TNF-α) and boosted IL-10. These data indicate that, in addition to the cytosolic effects of succinate on PHDs, its oxidation in the mitochondria is of vital importance for proinflammatory macrophage activation. This notion is supported by a recent report, showing that the recognition of bacteria induces changes in the architecture of the ETC [62]. When macrophages were incubated with living bacteria, the abundance of complex I-associated structures decreased, while complex II activity was stimulated. Interestingly, treatment with heat-inactivated bacteria did not evoke this response to the same extent, strongly suggesting that pathogen viability plays a vital role regarding the intensity of the induced immune response. It was further shown that LPS-stimulated macrophages mainly rely on glycolysis for ATP generation and, as a consequence, exhibit a higher mitochondrial membrane potential (ΔΨ_m_) [23]. This increase in ΔΨ_m_ emerges due to an accumulation of protons in the mitochondrial intermembrane space when the ATP synthase is inactive. When analyzing the impact of the increase in ΔΨ_m_ on macrophage activation, the authors found that its function lies in the production of mitochondrial ROS (mROS), which act as a signal to drive IL-1β production. mROS are most likely produced via reverse electron flow from complex II to complex I of the ETC, following succinate oxidation (Fig. 2) [63, 64]. Treatment with several ROS scavengers abolished LPS-induced mROS and significantly decreased IL-1β expression [23]. The same effect was observed in macrophages overexpressing alternative oxidase (AOX)—an enzyme which oxidizes excess electrons in the ubiquinone pool without affecting ΔΨ_m_. Strikingly, overexpression of AOX also increased the survival of mice injected with LPS [23]. The influence of increased mROS on IL-1β is likely mediated via the stabilization of HIF-1α, as was previously demonstrated [65]. These results are in line with a second study which showed a similar effect of complex I inhibition by metformin on both ROS generation and IL-1β production [66]. The fact that mROS and HIF-1α regulate the production of IL-1β, but not TNF-α, indicate a specificity of mitochondria regarding cytokine production. Of note, it was previously found that TLR signaling induces not only the production of mROS, but also the recruitment of mitochondria to phagosomes, indicating a bactericidal role of mROS [67]. Furthermore, mROS was shown to modify cytokine production by preventing the dephosphorylation of mitogen-activated protein kinases (MAPKs) [68] and activating the NLRP3 inflammasome [69, 70], which is involved in the maturation of IL-1β. The various roles of mROS (and ROS in general) highlight the importance of the cellular redox state, which is directly coupled to the metabolic activity of a cell, for the course of the inflammatory process.

### Role of the aspartate-arginosuccinate shunt

Besides succinate, the TCA cycle intermediate malate was also found to accumulate at later stages of the inflammatory response (24 h after LPS stimulation) [54]. Intriguingly, a [U-^13^C]glutamine tracer-based approach revealed a considerably higher amount of ^13^C-atoms in succinate as compared to malate. These observations can only be explained by increased activity of a glutamine-independent pathway which also contributes to the malate pool [71]. Based on similarities of pool size ratios between unstimulated and M1 macrophages as well as the corresponding ^13^C-labeling patterns, the authors identified the aspartate-arginosuccinate shunt (AASS) as a source of malate [54]. The AASS connects the TCA cycle with the urea cycle and feeds the malate pool indirectly by producing fumarate. At the same time, nitric oxide can be produced from argininosuccinate via the NO cycle (Fig. 2). The significance of this pathway was highlighted by chemical inhibition of the AASS in M1 macrophages, which led to decreased NO and IL-6 production and iNOS expression as well as a normalization of glycolytic activity and mitochondrial respiratory function. The latter is most likely due to the decrease in NO, which has been shown to suppress mitochondrial respiration in dendritic cells [72].

### Pseudohypoxic metabolism of proinflammatory macrophages

The LPS-induced stabilization of HIF-1α by cytosolic succinate [15] and mROS [23], even under normoxic conditions, pushes cells into a state termed pseudohypoxia [60]. These findings led to the speculation that LPS-induced HIF-1α stabilization uncouples carbon flux from glycolytic pyruvate into the TCA cycle by inhibiting pyruvate dehydrogenase (PDH) activity. This posttranscriptional inhibition is carried out by the HIF-1α target pyruvate dehydrogenase kinase (PDK) which inactivates PDH by phosphorylation. A study by Meiser et al. set out to test this hypothesis and demonstrated that M(LPS) macrophages sustained PDH activity, although HIF-1α protein was stabilized [73]. Surprisingly, the expression of *Pdk1* in murine macrophages even decreased in response to LPS, eventually leading to a slight increase in pyruvate oxidation by PDH (Fig. 2). Inhibition of pyruvate entry into the TCA cycle via chemical inhibition of the mitochondrial pyruvate carrier 1 (MPC1) resulted in decreased expression levels of *iNOS*, *Irg1*, and *Tnfα*, as well as lower intracellular levels of itaconic acid. The expression of IL-1β was only slightly lowered following MPC1 inhibition. These results highlight the fact that M(LPS) macrophages rely on mitochondrial pyruvate oxidation for the production of key cytokines and the synthesis of itaconic acid. The latter plays an important role during later stages of the immune response where it regulates proinflammatory cytokine production through inhibition of SDH [74, 75]. Activation of aerobic glycolysis through the action of HIF-1α and PDK was also found to play an essential role in macrophage mobilization and migration [76]. When PDK was inhibited by treatment with dichloroacetic acid (DCA), Semba et al. observed a decrease in cell migration, eventually leading to systemic inflammation in vivo [76].

Another metabolic feature commonly observed as a result of HIF-1α stabilization is the IDH-mediated, reductive carboxylation of α-ketoglutarate to isocitrate [77–79]. Under hypoxic conditions, where pyruvate entry into the TCA cycle is restricted, this reaction serves to produce citrate from glutamine to compensate for the decreased contribution from glucose. The generated citrate is then cleaved to produce cytosolic acetyl-CoA, which can be used for lipid synthesis. Interestingly, this metabolic phenotype was also not observed in M(LPS) macrophages, although glutamine uptake was increased as compared to resting macrophages [73]. It was shown that M(LPS) macrophages mainly rely on glucose-derived carbon for fatty acid synthesis, with the relative contribution from glucose to fatty acid carbons being around five times as high as the respective contribution from glutamine. These results point towards the existence of one or more yet unknown factors, which regulate the activities of PDK and reductive glutamine metabolism in LPS-activated macrophages. The importance of fatty acid synthesis is further highlighted by a study investigating the role of the mitochondrial citrate carrier—the first step in channeling citrate towards fatty acid synthesis [80]. The citrate carrier was found to be upregulated upon LPS stimulation and silencing or inhibition of the carrier reduced the production of nitric oxide, ROS, and prostaglandin E_2_.

### Protein succinylation

Succinate can further interfere with cellular metabolism via the succinylation of protein lysine residues (Fig. 2). Proteins that have been shown to undergo succinylation include important metabolic enzymes, including aspartate aminotransferase, glutamate dehydrogenase, malate dehydrogenase, and citrate synthase [81]. The degree of protein lysine succinylation is mainly regulated by the action of the NAD^+^-dependent desuccinylase and demalonylase SIRT5 [82, 83]. The expression of this enzyme was decreased in LPS-stimulated macrophages, while protein succinylation was doubled [15]. Both SIRT5 expression and global protein succinylation could be inhibited by treatment with 2-DG in a dose-dependent manner. Furthermore, LPS treatment led to a decrease in the NAD/NADH ratio, which might reflect limited availability of NAD^+^ for SIRT5-mediated protein succinylation. The shift towards NAD^+^ also provides further proof of the overall metabolic phenotype of CAMs with high glycolytic and low OxPhos activity. One recently discovered target of SIRT5 is PKM2 [84]. It was shown that SIRT5 activates PKM2 via desuccinylation in LPS-primed macrophages to boost the expression of IL-1β (also see section “Pyruvate kinase”). Further pathways affected by SIRT5-mediated protein desuccinylation include fatty acid β-oxidation and the synthesis of ketone bodies [85]. These findings clearly suggest a regulatory role for protein succinylation during the innate immune response which needs to be further investigated.

### Succinate as a signaling metabolite

Apart from its intracellular functions, succinate can also act as an autocrine and paracrine signaling metabolite [86]. Succinate can either be released into the extracellular milieu by damaged or necrotic cells [87], or when intracellular succinate accumulates—as for example during inhibition of SDH [88]. Two scenarios potentially observed during proinflammatory macrophage activation in vivo. Extracellular succinate is recognized by the G protein-coupled receptor GPR91 (also referred to as SUCNR1), which was found to be expressed on the cell surface of mature dendritic cells and macrophages (Fig. 2) [89, 90]. Its activation, in combination with TLR3 or TLR7 activation, augmented expression of the proinflammatory cytokines IL-1β and, interestingly, also TNF-α [90]. A recent study demonstrated a GPR91/succinate-dependent feed-forward loop of macrophage activation, ultimately leading to increased production of IL-1β [86]. Disturbance of this mechanism, e.g., by administration of GPR91 antagonists, was proposed as a novel approach to treat patients with rheumatoid arthritis.

## Itaconic acid

### Inhibition of bacterial growth

Another very exciting metabolite in the context of proinflammatory macrophage activation is itaconic acid (or methylenesuccinic acid). This metabolite was mainly believed to be a microbial metabolite until 2011, when Strelko and colleagues reported on the production of itaconic acid in activated macrophage cell lines and primary macrophages [91]. Two years later, immune-responsive Gene 1 (*Irg1*) was identified as the gene encoding the *cis*-aconitate decarboxylase (CAD) that catalyzes itaconic acid production from the TCA cycle intermediate *cis*-aconitate (Fig. 2) [92]. *Irg1* protein expression is massively potentiated in macrophages subjected to LPS or Th1 cytokines and is regulated by the interferon regulatory factor 1 (IRF1) [93]. At the time, the primary function of itaconic acid was suspected to be the inhibition of bacterial growth for microorganisms feeding on acetate or fatty acids [94]. This effect is mediated by itaconic acid inhibiting the isocitrate lyase (ICL) enzyme of the glyoxylate shunt [95]—a pathway absent in mammalian cells. In *Mycobacterium tuberculosis*, the ICL enzyme was further described to exhibit methylisocitrate lyase (MCL) activity. Methylisocitrate is an intermediate of the 2-methylcitrate cycle [96]—a pathway required for the detoxification of propionyl-CoA produced by the degradation of cholesterol from host macrophages [97]. This side-activity of ICL was also inhibited by the action of itaconic acid, confirming ICL as one of its targets [92]. To achieve itaconic acid concentrations that are sufficient to effectively inhibit bacterial growth, macrophages accumulate high amounts of the metabolite, most likely in the cytosol and/or phagolysosomes. It was previously shown that the *Irg1* protein associates with mitochondria [98]; however, it is still not clear where exactly itaconic acid is produced. One possibility is that itaconic is produced inside the mitochondria and then transported to the cytosol or the phagolysosomes via one of the numerous mitochondrial carriers. A recent study supports this notion by showing that CAD-containing mitochondria closely associate with *Legionella*-containing vacuoles in murine BMDMs [99]. This might hint towards a directed trafficking of itaconic acid to the location where it is required for the inhibition of bacterial growth. Alternatively, CAD might be localized at the outer mitochondrial membrane with its catalytic activity directly in the cytosol.

### Inhibition of SDH

Although the anti-microbial activity of itaconic acid has been well established, more recent studies have demonstrated that the role of itaconic acid in the context of inflammation seems to go far beyond inhibiting bacterial growth. It was shown that, in addition to inhibiting bacterial ICL, itaconic acid also acts as a competitive inhibitor of succinate dehydrogenase (SDH or complex II of the mitochondrial electron transport chain) in LPS-activated macrophages (Fig. 2) [74, 75]. Theoretically, itaconic acid could also directly contribute to the intracellular succinate pool by interconversion of the two metabolites, as was previously described for *Pseudomonas* sp. [100] and isolated liver mitochondria [101]. This possibility was elegantly excluded by applying a ^13^C stable isotope labeling approach, showing that, in murine macrophages, carbon atoms from itaconic acid do not end up in succinate [74]. Macrophages isolated from *Irg1*^−/−^ mice accumulated succinate to a lower extent as compared to their wild-type counterparts when challenged with LPS. Accordingly, inhibition of SDH by itaconic acid seems to be partially responsible for the accumulation of succinate observed in CAMs and thus plays a crucial role in governing the inflammatory response by regulating HIF-1α stabilization [15, 23]. Indeed, the administration of dimethyl itaconate, a potentially membrane permeable non-ionic form of itaconic acid, drastically suppressed the expression of the proinflammatory markers iNOS, IL-6, IL-18, IL-1β, and IL-12p70 [75]. The levels of TNFα remained unchanged, which is in accordance with the previous work on the influence of SDH and succinate on cytokine production [15, 23]. In contrast, ablation of *Irg1* had an amplifying effect on the production of the cytokines and inflammatory markers mentioned above (but not TNFα). Mechanistically, itaconic acid-mediated SDH inhibition might lead to the observed decrease in SDH-derived mitochondrial ROS (mROS), which have been reported to affect inflammasome priming [102]. The authors of that study could further show that the abundance of HIF-1α protein is increased in the absence of itaconic acid and decreased when exogenous itaconate is added. These observations indicate that the stabilization of HIF-1α and the subsequent promotion of IL-1β expression might rather be mediated by the inhibition of SDH activity than signaling through accumulating succinate. Either way, the influence of *Irg1* and itaconic acid on cytokine production clearly places them on the map as vital anti-inflammatory players, regulating the innate immune response.

### Metabolic reprogramming assists in itaconic acid production

In addition to the metabolic rewiring caused by the action of itaconic acid itself, another metabolic switch potentially supports itaconic acid production. Using a combined system approach of metabolomics and transcriptomics data, Jha et al. identified transcriptional downregulation of isocitrate dehydrogenase 1 (IDH1) and the subsequent disruption of TCA cycle flux as a major feature of M1 polarization (Fig. 2) [54]. This breakpoint at the site of IDH led to an accumulation of citrate and isocitrate, which represent the indirect precursors of itaconic acid. Based on ^13^C labeling data, no carbon atoms from glucose were passed on to α-ketoglutarate in M1 macrophages. Instead, glucose-derived carbon flux was redirected towards itaconic acid production, suggesting that glucose is an important carbon source for itaconic acid. However, only one out of the five carbon atoms of itaconic acid is derived from glycolytic acetyl-CoA. The remaining four carbons are derived from oxaloacetate, which can be produced from glutamine via glutaminolysis, or from glucose via the pyruvate carboxylase reaction. In zebrafish, it was recently shown that fatty acid oxidation for the production of mitochondrial ROS is dependent on the presence of CAD protein [103]. However, a role for fatty acids as a carbon source for itaconic acid production seems rather unlikely, given the highly glycolytic nature of M1 polarized macrophages. Meiser et al. analyzed which carbon sources contribute to itaconic acid production in RAW 264.7 cells and found that 27 and 59% of itaconic acid carbon atoms are derived from glucose and glutamine, respectively [73]. However, the carbon sources for itaconic acid production are likely dependent on the activity of other metabolic enzymes such as IDH and SDH and might, thus, change during the course of an infection.

The decrease in oxidative TCA cycle metabolism resulting from IDH1 downregulation [54] presumably contributes to the decrease in respirational activity, commonly observed in M1 macrophages [6]. Given the fact that these experiments were only performed after 20–24 h of stimulation and that the reduced activity of IDH1 is due to a decrease in its transcription, IDH1 might still be active during the early stages of infection. It would be interesting to get a more detailed picture of the dynamics of IDH1 inactivation during the course of an inflammatory response, since itaconic acid peaks around 10 h after stimulation [92]. Such experiments could help to shed light on the influence of IDH1 on itaconic acid production. Another open question concerns the IDH isoforms involved in this process. In their study, Jha et al. only indicate a role for the cytosolic isoform IDH1, although an involvement of the mitochondrial IDH2 seems more intuitive based on the observed implications for TCA cycle activity. Regardless of the isoform involved, the simultaneous decrease of both IDH and SDH activity during M1 polarization seems to play a vital role in controlling the inflammatory response in murine macrophages. Nonetheless, future investigations will have to show how these findings translate to other cell types and species.

### Are itaconic acid and dimethyl itaconate suited for investigating the intracellular mode of action of itaconic acid?

One recently published article adds a bitter taste to the results obtained from experiments where itaconic acid or dimethyl itaconate used to study the mechanistic effects of itaconic acid on cellular metabolism. ElAzzouny and colleagues synthesized ^13^C-labeled variants of both substances and demonstrated that itaconic acid is not taken up, and dimethyl itaconate is not metabolized by bone marrow-derived macrophages [104]. Remarkably, exogenous itaconic acid nonetheless managed to boost intracellular succinate levels. This would suggest that the effects observed in this and previous studies [74, 75] are not mediated through direct inhibition of SDH by itaconic acid, but rather through its binding to cell surface receptors. One potential candidate is the succinate receptor GPR91, given the structural similarity between succinate and itaconate [86]. The observed inconsistencies regarding the uptake of itaconic acid and the metabolization of dimethyl itaconate could potentially also be explained by differences in the experimental setup of the various studies (e.g., incubation times). However, if it holds true that itaconic acid and dimethyl itaconate are not suitable agents to increase intracellular itaconate levels, the previous studies will have to be re-interpreted accordingly.

### Inhibition of substrate-level phosphorylation

Another mode of action which was recently described for itaconic acid is the abolition of mitochondrial substrate-level phosphorylation (SLP, Fig. 2) [105]. During SLP, ATP or GTP is generated by a direct transfer of phosphate groups from phosphorylated intermediates to ADP or GDP, respectively. In mitochondria, SLP is mainly occurs during the succinate-CoA ligase reaction. This reaction becomes especially important under conditions where ATP generation via oxidative phosphorylation is impaired. Treatment of isolated liver mitochondria with itaconic acid dose-dependently inhibited and even reversed the directionality of the adenine nucleotide translocator, suggesting SLP inhibition. How the inhibitory effect of itaconic acid on SLP affects the metabolism and polarization state of macrophages, however, remains unclear. Given the inhibitory effect of itaconic acid on SDH, a component of the mitochondrial ETC, SLP inhibition seems counterintuitive, since it would make it harder for the already challenged macrophage to ensure sufficient energy generation. There could, however, be a benefit during infection when looking at the effect of SLP inhibition in the infective organism: succinate-CoA ligase-mediated SLP is essential for the survival of procyclic *Trypanosoma brucei* and potentially other microorganisms [106]. Besides the well-established inhibition of ICL, inhibition of SLP could be a second target for itaconic acid to impede microbial growth.

## Glutathione

Given the high rate of ROS production during classical activation, macrophages need a means to control and prevent excessive radical formation, which would otherwise lead to chronic inflammation. The main antioxidant used for this purpose is the tripeptide GSH (γ-l-glutamyl-l-cysteinyl-glycine). GSH can reach intracellular concentrations of up to 10 mM and plays an important role in maintaining cellular redox homeostasis [107, 108]. On the molecular level, ROS oxidize redox-sensitive cysteine residues in proteins to sulfenic, sulfinic, and sulfonic acid groups, thereby modifying protein tertiary structure and activity [109]. For sulfenic and sulfinic acid residues, this process can be reversed by GSH in combination with a respective reductase enzyme. Furthermore, GSH can be covalently bound to protein cysteine residues when ROS are present, as was recently shown for peroxiredoxin-2 [110] and the transcription factor HIF-1α [111]. In the case of HIF-1α, glutathionylation led to its stabilization. The deglutathionylation process is catalyzed by the glutaredoxin-1 enzyme, whose deletion increased HIF-1α activity and positively influenced ischemic revascularization. Modulation of HIF-1α activity via glutathionylation might also have significant implications for macrophage activation, as HIF-1α is involved in orchestrating the metabolic response of proinflammatory macrophages [15, 23, 34]. One especially interesting enzyme regarding glutathionylation in the context on inflammation is glutathione transferase omega 1 (GSTO1). Knockdown of this enzyme in macrophages was followed by a decrease in NOX1 expression and ROS production upon LPS stimulation [112]. GSTO1-deficient macrophages did not switch to the highly glycolytic phenotype usually observed in M(LPS) macrophages, possibly due to decreased levels of HIF-1α protein [113]. Accumulation of the immunometabolite succinate was also blocked in GSTO1-deficient cells. These results suggest a role for GSTO1 in the LPS-TLR4 proinflammatory pathway and make GSTO1 and GSH metabolism attractive targets for potential pharmacological interventions to treat chronic inflammation.

Another strategy in which macrophages use GSH to protect themselves from oxidative damage was recently unveiled. Lok et al. reported on a storage and transport system for NO, which helps macrophages in delivering NO to tumor cells without suffering from its cytotoxic effects themselves [114]. To this end, NO is sequestered as a dinitrosyl-dithiol iron complex (DNIC) by the glutathione *S*-transferase P1 (GSTP1) enzyme. In contrast to NO, DNICs do not induce cell death via the release of iron via the MRP1 transporter. In activated macrophages, both GSTP1 and MRP1 were upregulated and MRP1 was shown to mediate the export of DNICs. Decreased expression of GSTP1 and MRP1 was associated with NO-mediated cytotoxicity, strongly suggesting that this storage and transport system plays a vital role for macrophages in protecting themselves from the effects of their own RNS.

The importance of GSH for immunological processes is further underlined by a recent study which indentified the catalytic subunit of glutamate cysteine ligase (GCLc), the rate-limiting enzyme in GSH synthesis, as a critical component during T cell activation [115]. While GCLc-deficient T cells still exhibited normal, early activation, they were not able to reprogram their metabolism and proliferate upon activation. Ablation of the *Gclc* gene successfully prevented auto-immunity but also impaired antiviral defense in vivo.

## Branched-chain aminotransferase 1

Branched-chain aminotransferases (BCATs) are enzymes that convert the branched-chain amino acids valine, leucine, and isoleucine into the respective branched-chain ketoacids (BCKAs). BCAT1 was previously found to be highly expressed in tumors carrying non-mutated IDH1/2 alleles, where it promoted tumor cell proliferation and invasiveness [116, 117]. The function of BCATs in inflammation was, however, unknown. A recent study reported that human primary macrophages predominantly express cytosolic BCAT1 and that chemical inhibition of this isoform results in decreased oxygen consumption and glycolysis [118]. BCAT1 inhibition also downregulated the expression of *Irg1* and, in turn, itaconic acid production in M(LPS) macrophages. The mRNA and protein levels of both IL-1β and HIF-1α were not affected by these metabolic changes during early activation (3 h after LPS treatment), but IL-1β protein levels were significantly decreased later on (24 h after LPS treatment). These findings highlight the importance of choosing the correct timepoint when performing research on the biochemistry of macrophage activation. As an example, *Irg1* expression peaks at 6 h after LPS stimulation, whereas itaconic acid levels reach their maximum after 8–10 h [74, 92]. Depending on the underlying biological question, the timing of the experiment will have a crucial effect on the obtained result.

It will be interesting to see, if and how BCAT1 affects metabolic features of activated macrophages, especially the accumulation of succinate, SDH activity, and the production of mROS. Administration of the selective BCAT1 inhibitor ERG240 (a leucine analogue) to mice resulted in reduced severity of immune-mediated inflammation in vivo, indicating that BCAT1 inhibition could also be of potential therapeutic interest [118]. A second study on the role of BCAT1 in macrophages found that glioblastoma cells secrete BCKAs via the monocarboxylate carrier 1 (MCT1) and that these BCKAs are taken up by tumor-associated macrophages [119]. BCKA uptake is potentially mediated by the MCT1 and/or MCT4 carriers, both of which are expressed in macrophages. Subsequently, the BCKAs are aminated via BCAT1 to form either valine, leucine, or isoleucine (Fig. 2). Although a ^13^C tracing approach revealed no contribution of BCKAs to TCA cycle intermediates, macrophages treated with BCKAs exhibited impaired phagocytic capacity. Thus, these results identify BCKAs as a means of cancer cells to suppress immune function and sustain tumor growth.

## Prevention of chronic inflammation by the action of IL-10

An interesting study recently investigated the role of IL-10 in alleviating an ongoing inflammatory response [120]. IL-10 is an anti-inflammatory cytokine, which is produced by activated immune cells and sensed via the IL-10 receptor (IL-10R) [121]. Although IL-10R is expressed by many immune cells, macrophages have been shown to be the primary target of IL-10 [122, 123]. Mutations in either IL-10 or its receptor have been associated with chronic inflammation, underlining the importance of IL-10 signaling in regulating the immune response. On the metabolic level, macrophages from IL-10^−/−^ mice exhibited higher glycolytic rates, but decreased OxPhos activity as compared to wild-type macrophages, and both effects could be rescued by the addition of exogenous IL-10 [120]. The inhibitory effect of IL-10 on glycolysis was shown to be mediated by downregulation of glycolytic genes as well as the prevention of GLUT1 translocation from intracellular vesicles to the plasma membrane. IL-10^−/−^ cells further accumulated ROS-producing mitochondria with a loss of ΔΨ_m_. In wild-type cells, such mitochondria are usually subjected to mitophagy, as excessive ROS can amplify an inflammatory response in various ways (see section “Hexokinase” for more details). The observed effects seem to be mediated by IL-10 induced inhibition of mTORC1 via STAT3 and DDIT4. mTORC1 acts as a driver of glucose and lipid metabolism as well as an inhibitor of autophagy [124]. Upon TLR4 activation, mTORC1 is initially active to allow for proinflammatory metabolic reprogramming with high glycolytic rates and mitochondrial ROS production [15, 23, 125]. In IL-10^−/−^ macrophages, mTORC1 signaling is more persistent, which eventually pushes macrophages towards an uncontrolled, proinflammatory state. Interestingly, the IL-10 induced expression of DDIT4 is independent of HIF-1α, a known regulator of DDIT4 in hypoxia [126].

## NOX4 and fatty acid oxidation

NADPH oxidases are one of the main sources for ROS in activated macrophages [42, 43] and as such they have a significant impact on the course of an inflammatory response. Besides its ROS-generating function, the mitochondrial NOX4 isoform was also found to control fatty acid β-oxidation [127]. Knockout of NOX4 reduced the expression of carnitine palmitoyltransferase 1A (CPT1A). This enzyme catalyzes the transfer of a fatty acid acyl-group from coenzyme A to carnitine—a crucial step for the initiation of fatty acid oxidation. The concomitant decrease in fatty acid oxidation resulted in decreased NLRP3 inflammasome activation and reduced production of the proinflammatory cytokines IL-1β and IL-18 [127]. A similar effect was observed when treating BMDMs with the CPT1 inhibitor etomoxir. Ablation of NOX4 in a mouse model led to improved survival during NLRP3-mediated *Streptococcus pneumoniae* infection. These results suggest that fatty acid oxidation acts as a driver of proinflammatory activation by promoting NLRP3 inflammasome activation. This finding came as a surprise, given that CPT1A was previously found to be necessary for the polarization of alternatively activated M[IL-4] macrophages [128]. Accordingly, fatty acid oxidation seems to take different roles, depending on the cellular context. One additional factor which should be taken into account is that results obtained from studies using etomoxir should be interpreted with caution. A recent study showed that etomoxir exerted the same effect on M2 polarization in both wild-type and CPT2-deficient BMDMs [129]. This clearly demonstrates that the effects of this inhibitor on M2 polarization are caused by yet unknown mechanisms which are independent of CPT inhibition.

## Summary

The increasing number of studies on immunometabolism clearly reflects the importance of metabolism as a critical modulator of inflammation. Recent studies have shown that metabolism reprogramming is a key prerequisite for proper macrophage function, rather than merely a side effect of macrophage activation. Since metabolism is highly dynamic, it provides the perfect basis to allow for a swift response to environmental changes, which is especially important for the adaptation to pathogen infection. This adaptation is governed by the interplay of many metabolic enzymes, some of which exhibit previously unknown functions to drive inflammation. For example, GAPDH was shown to bind to and repress the translation of TNFα mRNA [25]. Both the complex interactions between numerous metabolic enzymes, pathways, and metabolites as well as changes in their behavior over the course of the activation process make this topic very challenging to investigate [130].

Notably, a large number of the metabolic features found in proinflammatory macrophages are also observed in cancer cells. This does not come as a surprise, given the fact that both cell types face demanding conditions with the need for high amounts of energy to support either immune functions or rapid growth. Thus, the most striking similarities are found in the way which their energy metabolism is rearranged: both cell types mainly rely on aerobic glycolysis for energy generation and exhibit low rates of mitochondrial OxPhos. In some cases, activated macrophages and cancer cells even exploit the same regulatory mechanisms to reach their goals, as was shown, e.g, for the switch to PKM2 [34], stabilization of HIF-1α [15, 131], or expression of BCAT1 [118].

One of the most remarkable metabolic changes in activated macrophages is the CAD-mediated production of itaconic acid. This metabolite stands out, as its production in mammals seems to be confined to cells of macrophage-lineage [132]. Even though an increasing number of studies on the role and importance of *Irg1* and itaconic acid are currently being published, some open questions remain. One exciting topic is the intracellular compartmentalization and trafficking of itaconic acid: Is itaconic acid produced by the mitochondria or in the cytosol, and which carriers mediate its transport between these two compartments and phagolysosomes? Newly developed methods could help to address these questions [133]. Another question to be answered is: Is itaconic acid degraded, or simply secreted from the cell, as some studies suggest [75, 91]? If the latter is the case, could it potentially be used as a biomarker for infectious diseases? What happens to itaconic acid once the proinflammatory response is over? And lastly: Are there still unknown functions of itaconic acid that are waiting to be unveiled? A recent study found that several cell lines, including a murine macrophage line, produce itaconyl-CoA from itaconic acid [134]. Accumulating itaconyl-CoA was identified as a cofactor-inactivating inhibitor of the mitochondrial B_12_-dependent methylmalonyl-CoA mutase (MUT). This enzyme plays a vital role in the catabolism of odd chain fatty acids and branched-chain amino acids. If and how itaconyl-CoA-mediated MUT inhibition affects macrophage activation will need to be investigated in future studies.

In addition to the ways in which metabolism controls macrophage activation that are discussed in this review, metabolic activity and the accumulation of metabolic intermediates are also tightly coupled to epigenetic modifications [135, 136]. Metabolic products such as acetyl-CoA, α-ketoglutarate, fumarate, NAD^+^, or *S*-adenosylmethionine affect epigenetic modifications either by acting as substrates or indirectly by modulating the activity of epigenetic enzymes. These processes have recently been shown to have a strong impact on immunological processes such as trained immunity [137] and are an exciting field for further investigations.

The aim of all immunological research is to increase our understanding of the mechanisms involved the immune response to find new ways to treat or prevent diseases. In the case of macrophage activation, there are two directions which can potentially lead to novel therapeutic approaches: inducing a shift from M1 to M2 would be beneficial for the treatment of chronic and auto-immune diseases, whereas enhancement of the M1 phenotype could help cope with infectious diseases. Metabolic intervention is a promising tool to achieve these goals, given the importance of metabolic reprogramming for macrophage activation. One major problem, however, is that metabolic targets are, in most cases, not specific to macrophages or immune cells, which can leads to off-target effects in other tissues. Furthermore, immune cells might respond differently to a given treatment, depending on the tissue environment they are facing. Future research will have to set out to overcome these problems to discover novel therapeutic approaches.
